# Phenotypic divergence in rotator cuff tear and volumetric muscle loss mouse models following fibroadipogenic progenitor depletion

**DOI:** 10.1302/2046-3758.158.BJR-2025-0112.R2

**Published:** 2026-08-01

**Authors:** Zili Wang, Luke Sang, Alex Youn, Mengyao Liu, Brian T. Feeley, Xuhui Liu

**Affiliations:** 1 Department of Orthopaedic Surgery, The Third Xiangya Hospital of Central South University, Changsha, China; 2 Department of Orthopaedic Surgery, University of California San Francisco, San Francisco, California, USA; 3 San Francisco Veteran Affairs Health Care System, San Francisco, California, USA; 4 School of Medicine, University of California San Francisco, San Francisco, California, USA

**Keywords:** Fibro-adipogenic progenitors, Musculoskeletal injury, Muscle regeneration, Rotator cuff tear, Volumetric muscle loss, rotator cuff tears (RCTs), mouse models, Fibroadipogenic progenitors, fibrosis, supraspinatus muscle, RCTs, Tamoxifen, muscle regeneration, muscle degeneration, shoulder

## Abstract

**Aims:**

Fibroadipogenic progenitors (FAPs) are a group of resident muscle stem cells capable of differentiating into fibroblasts and adipocytes, contributing to intramuscular fibrotic and fatty degeneration after injury. However, FAPs are also thought to play a crucial role in muscle regeneration by facilitating satellite cell myogenesis. Despite this dual role, the precise functions of FAPs in muscle degeneration and regeneration remain unclear. This study aimed to utilize a FAP depletion mouse model to define the role of FAPs in two distinct, clinically relevant injury models of rotator cuff tears (RCTs) and tibialis anterior (TA) volumetric muscle loss (VML).

**Methods:**

Six PDGFRα-Cre^ERT^/DTA mice were applied for fluorescence-activated cell sorting (FACS) evaluation of FAP depletion efficiency with tamoxifen induction. Then, two groups of ten PDGFRα-Cre^ERT^/DTA mice and five DTA mice underwent either unilateral supraspinatus and infraspinatus tendons and suprascapular nerve transection (RCT model) or the creation of a 4 mm diameter defect in the unilateral TA (VML model). To induce FAP depletion, tamoxifen or corn oil (control) was administered daily for two weeks before surgery. Gait analysis was conducted at six weeks post-surgery to evaluate shoulder or hindlimb function. Supraspinatus or TA muscles were harvested to assess muscle atrophy and for histological analysis.

**Results:**

FACS analysis confirmed a 50% reduction in FAPs following tamoxifen administration in PDGFRα-Cre^ERT^/DTA mice. In the RCT model, FAP depletion significantly attenuated muscle atrophy and improved fibrosis, fatty infiltration (FI), and global shoulder function. Conversely, in the VML model, FAP depletion significantly decreased muscle fibrosis but had no effect on FI and functional outcomes.

**Conclusion:**

Our results suggest that FAPs play distinct roles in muscle regeneration depending on the specific clinical context. These models can be used to further understand the different injury mechanisms and muscle-specific properties influencing FAP roles in musculoskeletal pathology.

Cite this article: *Bone Joint Res* 2026;15(8):927–936.

## Article focus

- The purpose of this study was to investigate the role of fibroadipogenic progenitors (FAPs) in two distinct, clinically relevant orthopaedic injury models (rotator cuff tears (RCTs) and tibialis anterior (TA) volumetric muscle loss (VML)) with an inducible FAP depletion mouse model.

## Key messages

- FAP depletion significantly attenuated muscle atrophy and improved fibrosis, fatty infiltration (FI), and global shoulder function after rotator cuff tears, but had minimal effects on muscle quality and functional outcomes after TA volumetric muscle loss.
- These findings suggest that FAPs play distinct roles in muscle regeneration depending on the specific clinical context, particularly across a chronic degenerative (RCT) versus an acute traumatic (VML) model in the TA.

## Strengths and limitations

- This study highlights the distinct roles of FAPs in two clinically relevant orthopaedic injury models.
- The inducible FAPs depletion mouse models used in this study enable time-sensitive depletion, allowing for the assessment of the role of those progenitor cells during muscle regeneration.
- Further research is needed to elucidate the mechanisms underlying different muscle injuries that contribute to the distinct roles of FAPs.

## Introduction

Fibroadipogenic progenitors (FAPs), identified by their expression of platelet-derived growth factor receptor α (PDGFRα), represent a population of resident muscle progenitor cells that play a pivotal role in responding to both direct and indirect muscle injuries. Under normal, healthy conditions, FAPs remain quiescent and reside within the interstitial spaces between myofibres.^1-3^ However, upon muscle injury, FAPs proliferate and actively contribute to muscle healing and regeneration.^2^ Notably, FAPs have also been shown to mediate degenerative processes, exhibiting a dual role in muscle biology.^2,4^ The mechanisms governing these opposing roles remain a significant focus of current research, with clinical context and chronicity emerging as critical determinants.^1,3,5-12^

Despite the growing recognition of FAPs in both degenerative and regenerative processes, the differences in how these progenitor cells influence muscle homeostasis in various musculoskeletal pathologies are still not fully understood. Recent studies have demonstrated the heterogeneous impact of FAPs across acute and chronic injuries and injury mechanisms.^13^ FAPs have been shown to be essential to myogenic stem cell differentiation and muscle regeneration in acute muscle injuries.^14^ Meanwhile, in chronic conditions, FAPs have been shown to differentiate into adipocytes and fibroblasts, contributing to deleterious changes.^4^ Further understanding of the functional role of FAPs in different injury models would provide crucial insights into amplifying or mitigating the effects of FAPs across various musculoskeletal pathologies.

Therefore, in this study, we applied two clinically relevant orthopaedic models to a FAP depletion model: rotator cuff tear (RCT) as a chronic degenerative injury, and tibialis anterior (TA) volumetric muscle loss (VML) as an acute muscle injury. By utilizing FAP depletion mice,^15^ our aim is to investigate the different impacts of FAPs on muscle regeneration and the subsequent response to both massive RCT and TA VML. Given the contrasting nature of these two injuries and clinical contexts, we hypothesize that FAP depletion will result in significant improvement in histological parameters, such as fatty infiltration (FI) and fibrosis, and functional outcomes in the RCT model, while FAP depletion will not result in these improvements in the TA VML context and will reduce muscle regeneration.

## Methods

### FAP depletion mouse model

An inducible FAP depletion mice model was generated by crossing B6N.Cg-Tg (Pdgfrα-cre/ERT)467Dbe/J (Stock #018280, Jackson Laboratory, USA) with B6.129P2-Gt (ROSA)26Sortm*^1(DTA)Lky/^*J (Stock #009669, Jackson Laboratory). Genotyping was performed to confirm the genotype of the PDGFRα-Cre^ERT(+)^/diphtheria toxin A (DTA) and PDGFRα-Cre^ERT(-)^/DTA mice. Both male and female mice were used in this study when they were three months old. All mice were maintained on a standard diet and environment with 12-hour light and dark cycles. To deplete FAPs, 80 mg/kg tamoxifen or corn oil (vehicle control) was administered for five days per week, for two weeks before surgery through intraperitoneal injections.

### FAP depletion efficiency

**Fig. 1:**
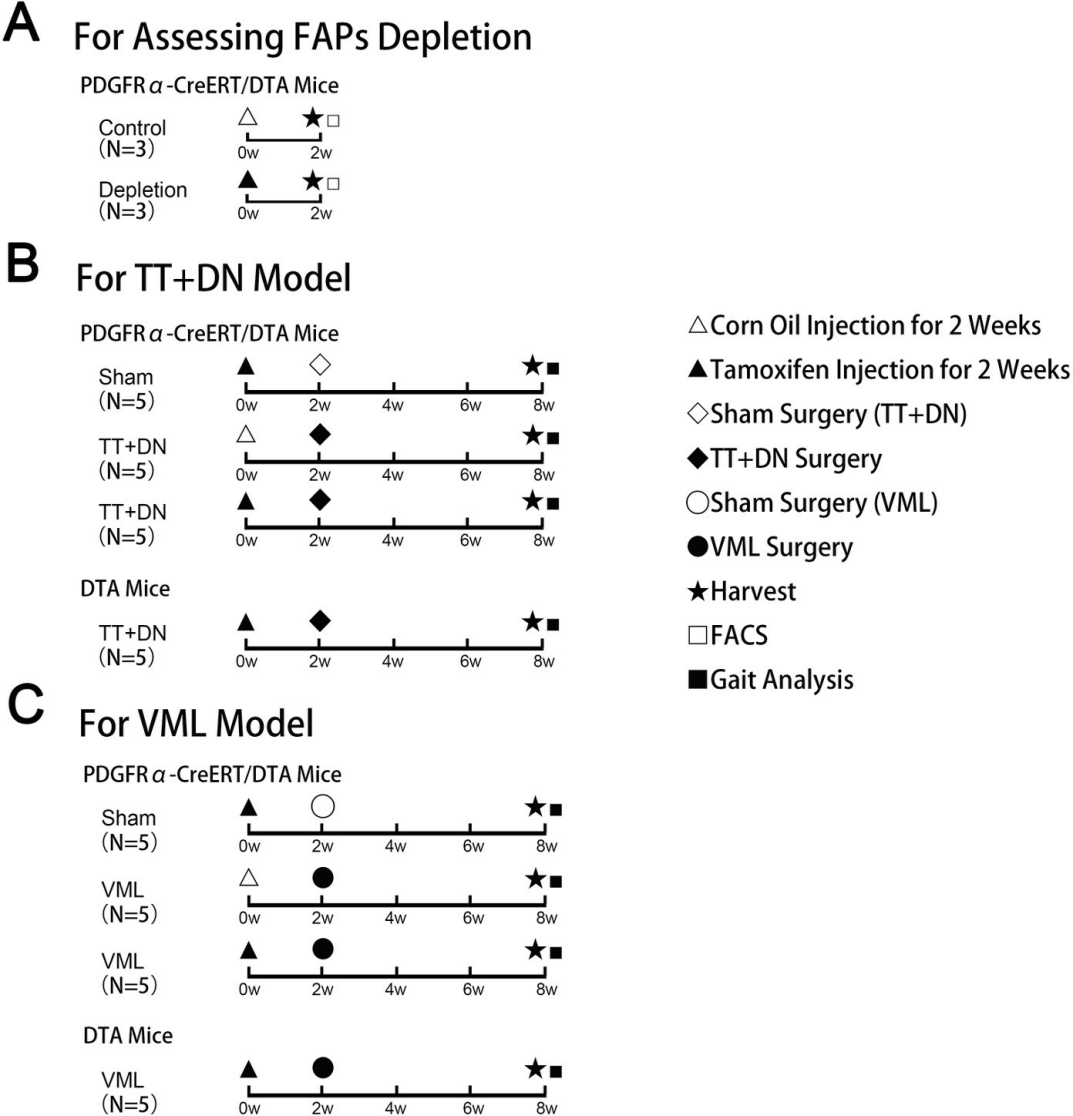
Flow diagram of experimental design. a) Six platelet-derived growth factor receptor α (PDGFRα)-CreERT/DTA mice were applied for evaluating fibroadipogenic progenitor (FAP) depletion efficiency. To deplete FAPs, tamoxifen was injected daily for two weeks before supraspinatus and tibialis anterior (TA) muscle harvest for fluorescence-activated cell sorting analysis. Corn oil injections were used as a vehicle control. b) Ten PDGFRα-CreERT/DTA mice and five DTA mice underwent unilateral supraspinatus and infraspinatus tendons and suprascapular nerve transection surgery (TT + DN) after two weeks of daily tamoxifen or corn oil injections. Five PDGFRα-CreERT/DTA mice also underwent sham surgery to serve as a control. Six weeks after injury, gait analysis of shoulder function was conducted, and the supraspinatus was harvested for muscle atrophy and histological analysis. c) The same distribution of mice and experimental timeline used in the TT + DN model was used for the volumetric muscle loss (VML) model. Gait analysis of hindlimb function was conducted six weeks after surgery, and the TA was harvested for muscle atrophy and histological analysis. FACS, fluorescence-activated cell sorting.

To confirm the depletion of FAPs in muscle in PDGFRα-Cre^ERT^/DTA mice, we conducted a pilot experiment (Figure 1a). Two weeks after tamoxifen or corn oil injections, three mice in each group were killed. Supraspinatus and TA muscles were subsequently harvested and digested, as described in detail below. FAPs were sorted and quantified using fluorescence-activated cell sorting (FACS). CD31-/ CD45-/ Integrin α7-/ Sca1+/ PDGFRα+ cell populations were counted as FAPs to calculate the percentage of cell number. Wet muscle weight loss and histological myofibre size analyses revealed no differences following tamoxifen injection compared to corn oil, confirming that FAP depletion had no baseline impact on myofibre health before injury (Supplementary Figures 1A to J).

### Surgical procedures

For RCT surgery, ten PDGFRα-Cre^ERT(+)^/DTA mice and five PDGFRα-Cre^ERT(-)^/DTA mice underwent unilateral supraspinatus and infraspinatus tendons and suprascapular nerve transection surgery (TT + DN), as described by Liu et al^16^ (Figure 1b). In brief, mice under general anaesthesia with 1% to 5% isoflurane underwent a complete unilateral supraspinatus tendon transection and suprascapular nerve transection to mimic a severe RCT. An additional five PDGFRα-Cre^ERT(+)^/DTA mice underwent sham surgery as a surgery control.

Similar to the RCT group, ten PDGFRα-Cre^ERT(+)^/DTA mice and five PDGFRα-Cre^ERT(-)^/DTA mice underwent unilateral TA VML surgery, as previously described (Figure 1c).^17^ In brief, under general anaesthesia with 1% to 5% isoflurane, the fur on the leg skin of the mice was shaved. After disinfection with betadine and 75% ethanol, an incision was made on the skin to expose the TA muscle. A VML was achieved by removing a portion of the TA muscle at full thickness with a 4 mm in diameter dermal puncture for all mice, resulting in approximately 30% loss of wet muscle weight.^18^ The wound was then covered with fascia and the skin was closed with sutures. Again, five PDGFRα-Cre^ERT(+)^/DTA mice underwent sham surgery as a control. All mice were treated with a post-surgery analgesic of one Buprenorphine 1.5 mg/kg subcutaneous injection. All experiments were approved by the Institutional Animal Care and Use Committee of the San Francisco VA Medical Center. We have adhered to the ARRIVE guidelines and have supplied the ARRIVE Checklist.

### Gait analysis

Gait analysis was conducted using DigiGait (Mouse Specifics, USA) to measure limb function at six weeks after surgery (forelimb for RCT model and hindlimb for VML model). During the gait testing, mice walked at the speed of 10 cm/s for 10 seconds on a horizontally leveled treadmill. Kinematic gait parameters, including stride length, stance width, maximum rate of acceleration change, and swing time, were collected and compared across surgical and sham groups. These parameters were specifically chosen to assess the degree of pain and motor ability, as reported in previous studies.^19-21^

### Muscle harvest, image capture, and histological quantification

Supraspinatus muscles (for the RCT model) or TA muscles (for the VML model) were harvested after mice were killed six weeks after the surgery. Wet muscle weight was measured, and relative muscle weight loss and degree of muscle mass recovery were interpreted in relation to the contralateral muscle weight: ([Injured muscle weight – contralateral control weight] / Contralateral control weight) × 100%.^22^ After wet weight measurements, muscle samples were then mounted on cork discs with 10% tragacanth gum (Thermo Fisher Scientific, USA; Product#11468417) in water and flash-frozen in liquid nitrogen-cooled isopentane (Thermo Fisher Scientific, Product#11410500) and sectioned at a thickness of 10 µm using a cryostat. For the supraspinatus muscle, the abdomen of the muscle was sectioned. For the TA muscle, the defect site was sectioned. Sections were then stained with Oil Red O (Sigma Aldrich, USA) to assess the degree of FI and Masson Trichrome (American MasterTech, USA) to evaluate the fibrosis severity in muscles, as previously described.^23^ Staining was conducted according to the manufacturer’s instructions. Slide images were captured with an optical microscope (Axio Imager; Zeiss, Germany), and these images were later analyzed using ImageJ (ImageJ, National Institutes of Health, USA) using macros to quantify myofibre size, the degree of fibrosis (fibrotic area / total muscle area × 100%), and FI (fat-infiltrated area / total muscle area × 100%).^24^

### Statistical analysis

One-way analysis of variance with Tukey’s post hoc tests was used for statistical analysis between experimental groups. Data are presented as mean (SD). Significance was defined as p < 0.05. All statistical analyses were calculated in PRISM GraphPad (GraphPad Software, USA).

## Results

### Tamoxifen induces reduction of FAP populations in supraspinatus and TA muscles of PDGFRα-Cre^ERT^ / DTA mice

**Fig. 2:**
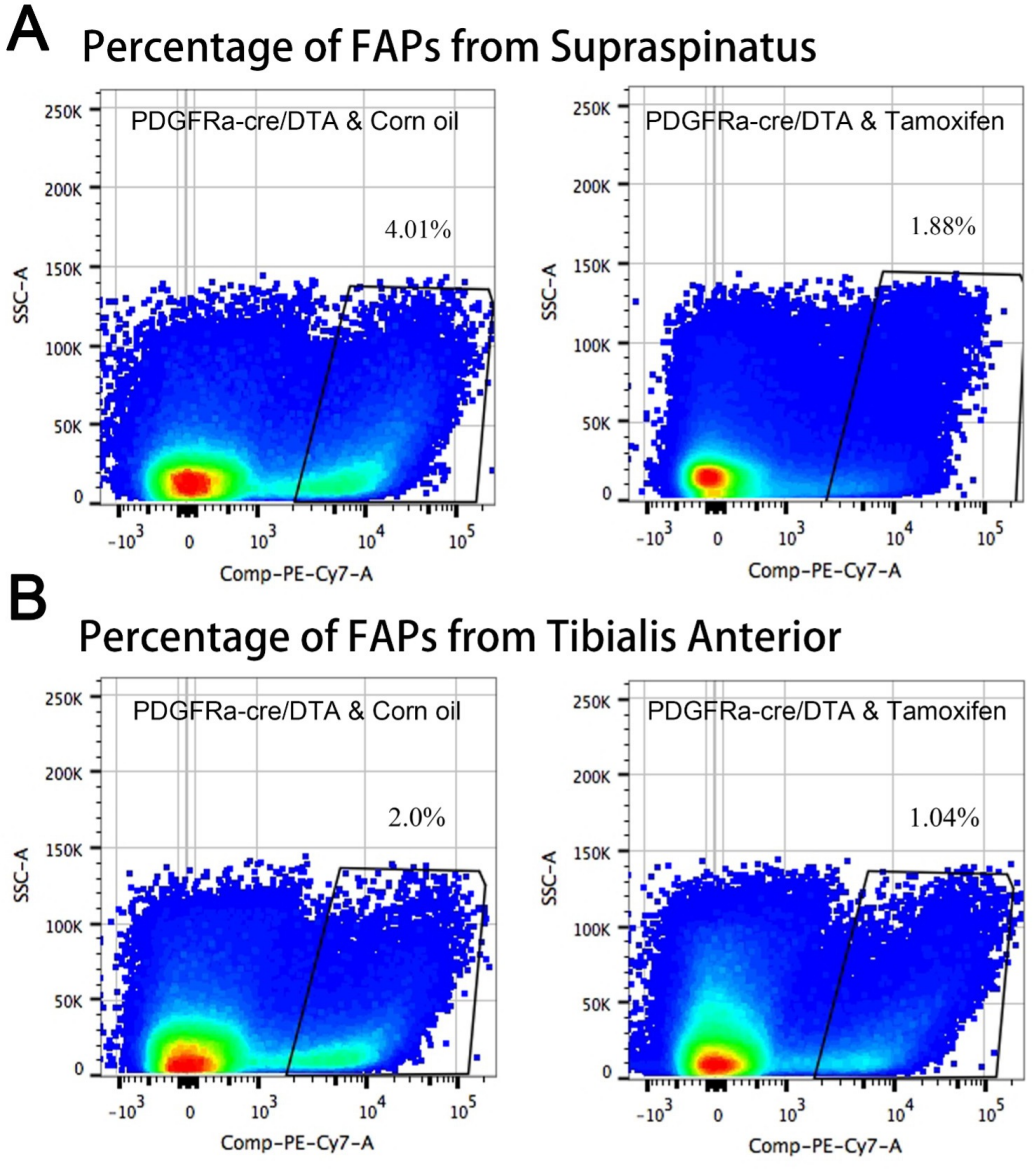
Murine fibroadipogenic progenitor (FAP) depletion efficiency data. a) Representative fluorescence-activated cell sorting plots demonstrating the efficiency of FAP depletion in supraspinatus and b) tibialis anterior (TA) muscles. CD31-/ CD45-/ Integrin α7-/ Sca1+/ PDGFRα+ cell population was counted as FAPs to calculate the percentage of cell number. Tamoxifen-injected mice had an approximately 50% reduction of FAPs in both the supraspinatus and TA muscles compared to the control (n = 3 mice per condition). SSC-A, side scatter area.

First, to determine the efficiency of our murine FAP depletion model, PDGFRα-Cre^ERT^/DTA mice were injected daily with either tamoxifen or corn oil, serving as a control, for two weeks prior to harvesting the supraspinatus and TA muscles. In the supraspinatus, FACS analysis revealed an approximately 50% reduction in the population percentage of FAPs for tamoxifen-treated mice compared to the control (1.88% vs 4.01%; Figure 2a). A similar effect was seen in the TA muscle when comparing the total percentage of FAPs in treated mice compared to control mice (1.04% vs 2.00%; Figure 2b). Immunofluorescence staining validation of FAP depletion in supraspinatus and TA muscle showed a significant decrease in PDGFRα-positive cells following tamoxifen treatment compared to corn oil control (Supplementary Figures 2A to E).

### Depletion of FAPs reduces supraspinatus fibrosis and FI after TT + DN

**Fig. 3:**
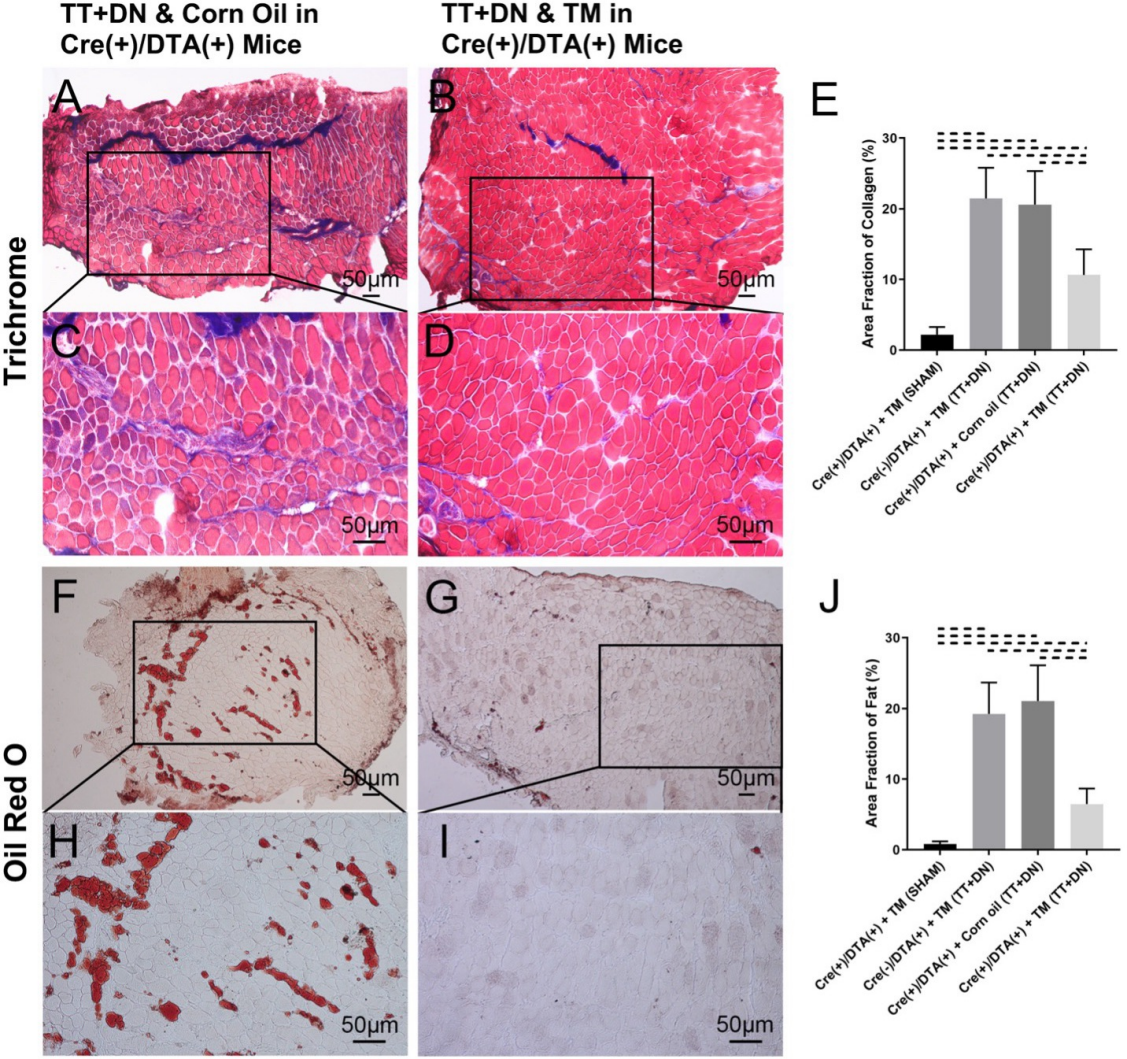
Supraspinatus intramuscular fibrosis and fatty infiltration (FI) following supraspinatus and infraspinatus tendons and suprascapular nerve transection surgery (TT + DN). a) to d) Representative trichrome staining images of supraspinatus muscle sections six weeks after TT + DN illustrating the degree of fibrosis. e) Depletion of fibroadipogenic progenitors (FAPs) showed significantly lower levels of fibrosis compared to the control (n = 5 mice per condition). f) to i) Representative Oil Red O staining images of post-TT+ DN supraspinatus muscle sections illustrating the degree of FI. j) There was a significant decrease in FI with FAP depletion compared to the control (n = 5 mice per condition). a), b), f), and g): ×100 magnification. c), d), h), and i): ×200 magnification. Data are presented as means. Error bars indicate the SD. Dashed lines indicate p < 0.01. TM, Tamoxifen.

Having established our model, we next examined the impact of FAP reduction on muscle degeneration after RCT. Histological analysis of the supraspinatus muscle six weeks post TT + DN surgery revealed significantly reduced fibrosis area in FAP-depleted mice compared to controls (10.64% (SD 3.61%) vs 20.60% (SD 4.73%), p = 0.003; Figures 3a to 3e). There was also a significant decrease in mean FI for the FAP depletion mice compared to the control (6.46% (SD 2.22%) vs 21.05% (SD 5.05%), p < 0.001; Figures 3f to 3j). By contrast, muscle harvested six weeks after sham surgery from FAP depletion mice showed minimal levels of fibrosis (2.19% (SD 1.08%); Figure 3e) and FI (0.82% (SD 0.38%); Figure 3j).

### FAP depletion attenuates muscle atrophy and improves global shoulder function after TT + DN

**Fig. 4:**
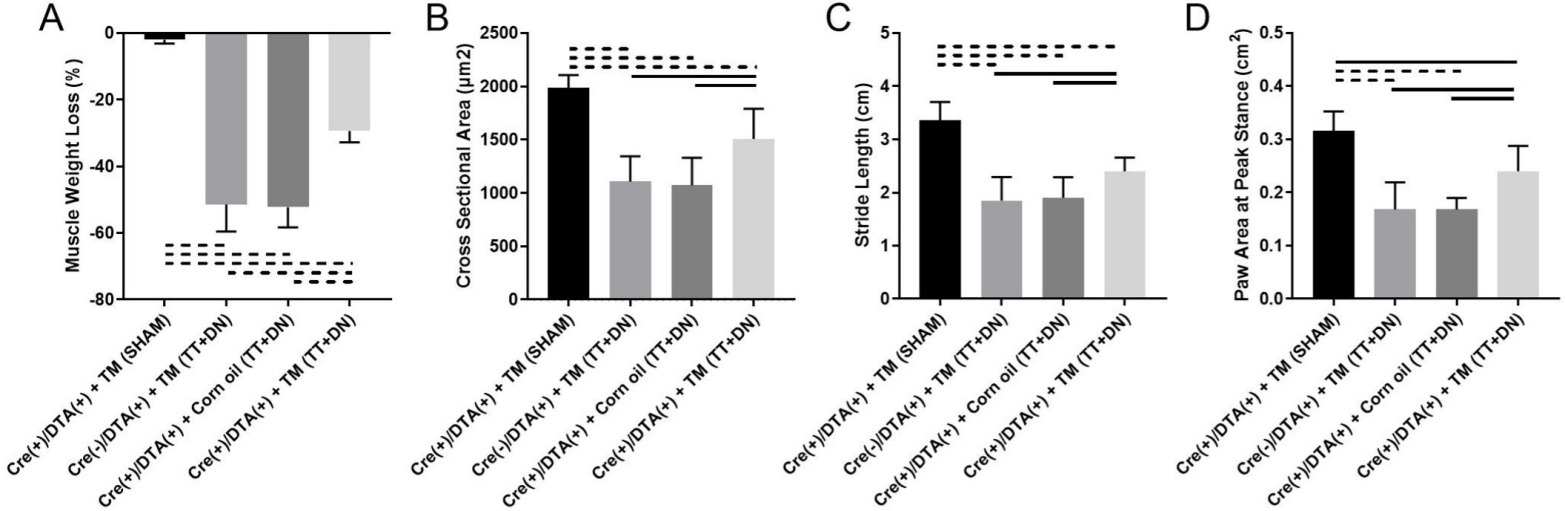
Supraspinatus muscle atrophy and shoulder functional analysis following supraspinatus and infraspinatus tendons and suprascapular nerve transection surgery (TT + DN). Depletion of fibroadipogenic progenitors (FAPs) significantly improved muscle atrophy after TT + DN compared to the control, as demonstrated by a) a significantly decreased loss of muscle weight and b) greater myofibre cross-sectional area (n = 5 mice per condition). c) and d) There were significantly improved shoulder function metrics with the depletion of FAPs compared to the control groups (n = 5 mice per condition). Data are presented as means. Error bars indicate the SD. Solid line indicates p < 0.05, and dashed lines indicate p < 0.01. TM, Tamoxifen.

Six weeks post-TT + DN, supraspinatus muscle weight loss was significantly reduced in FAP depletion mice compared to the control (-29.40% (SD 3.38%) vs -52.28% (SD 6.00%), p < 0.001; Figure 4a). There was a greater myofibre size in the FAP depletion group post-RCT compared to the control (Figure 4b). Additionally, FAP-depleted mice exhibited significantly improved shoulder function, as evidenced by increased mean stride length (2.40 cm (SD 0.26) vs 1.90 cm (SD 0.39), p = 0.045; Figure 4c) and mean paw area at peak stance (0.24 cm^2^ (SD 0.05) vs 0.17 cm^2^ (SD 0.02), p = 0.015; Figure 4d) compared to the control mice.

### Depletion of FAPs reduces TA fibrosis after VML

**Fig. 5:**
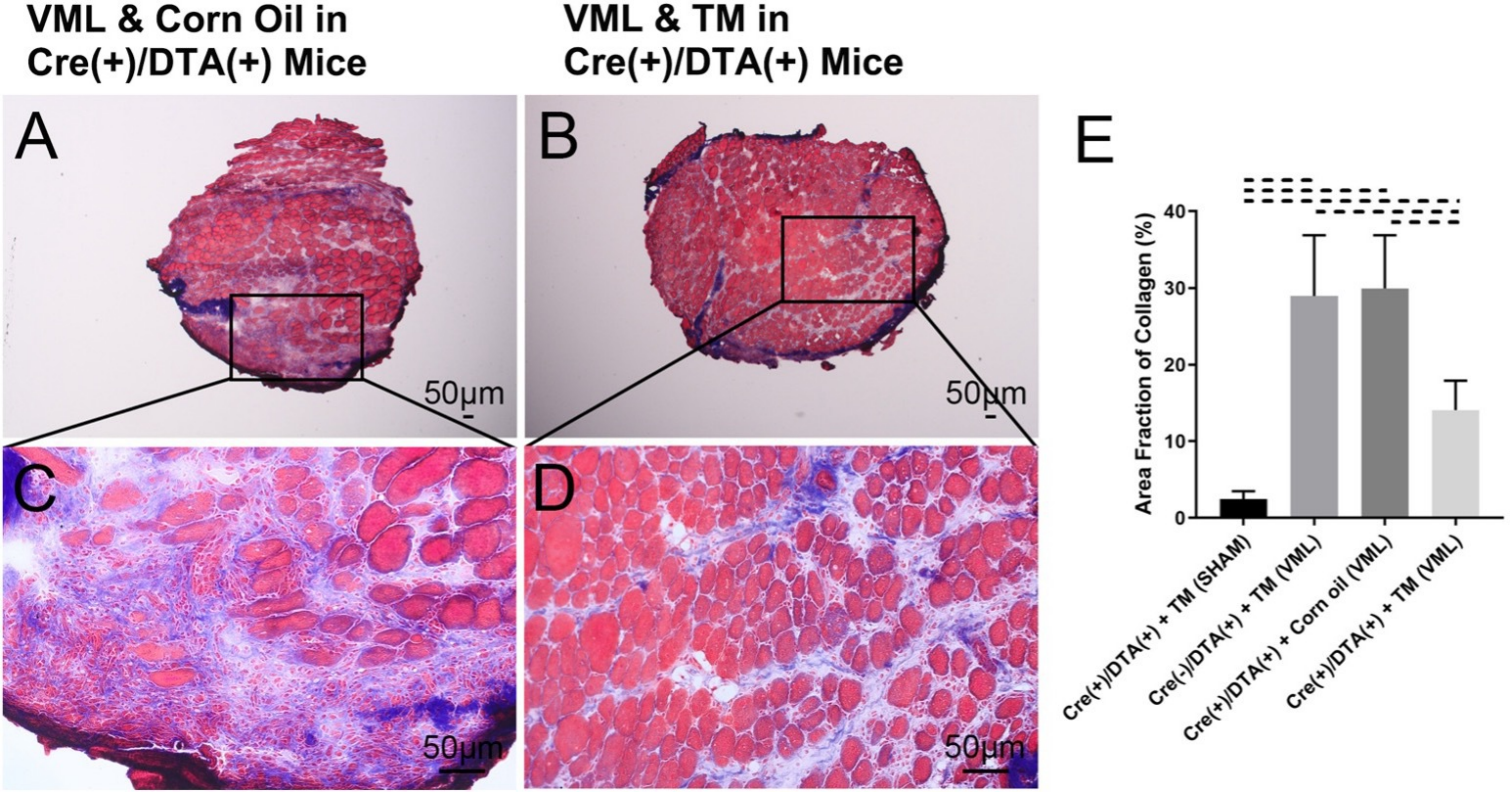
Tibialis anterior (TA) intramuscular fibrosis following volumetric muscle loss surgery (VML). a) to d) Representative trichrome staining images of TA muscle sections six weeks after VML illustrating the degree of fibrosis. e) The fibroadipogenic progenitors (FAPs) depletion group had significantly lower levels of fibrosis compared to the control groups (n = 5 mice per condition). a) and b) ×40 magnification. c) and d) ×200 magnification. Data are presented as means. Error bars indicate the SD. Dashed lines indicate p < 0.01. TM, Tamoxifen.

In the VML model, histological analysis of TA muscle six weeks post-injury revealed a significant mean reduction in fibrosis area in FAP-depleted mice compared to controls (14.06 ± 3.86% vs 29.95 ± 6.94%, p = 0.002; Figures 5a to 5e). In contrast, sham-operated mice exhibited minimal fibrosis (2.48 ± 1.00%, Figure 5e). Notably, no FI was observed in any group within the VML model.

### FAP depletion has no effect on muscle regeneration and hindlimb function after VML

**Fig. 6:**
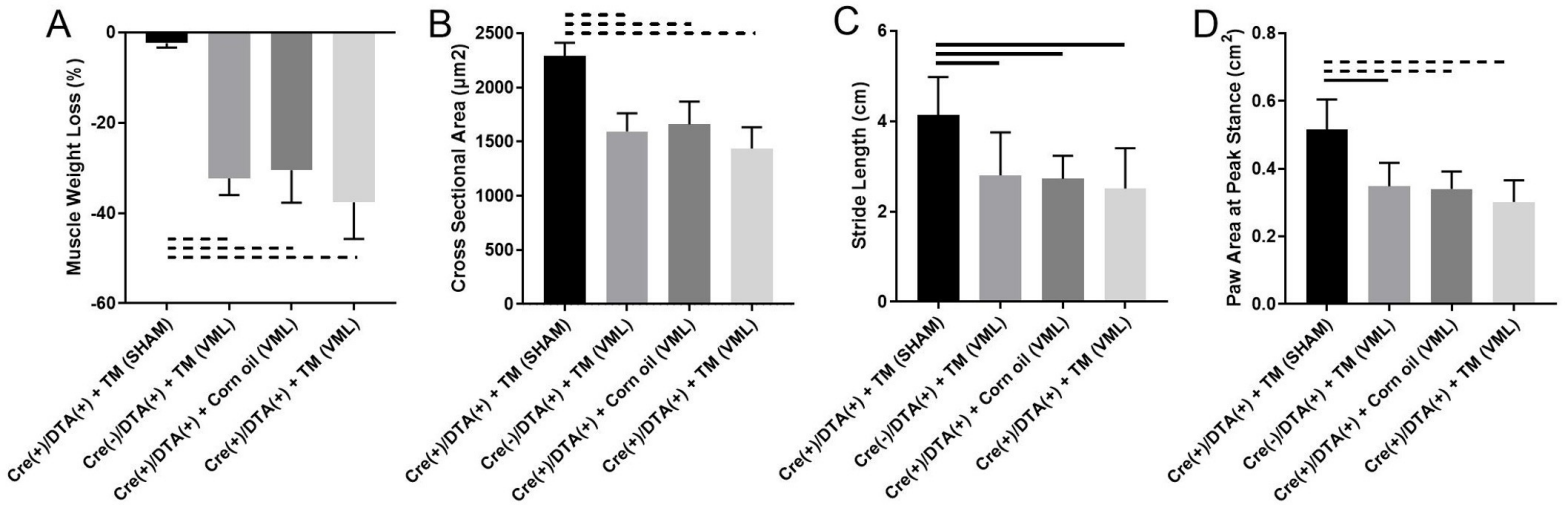
Tibialis anterior (TA) muscle atrophy and hindlimb functional analysis following volumetric muscle loss surgery (VML). Fibroadipogenic progenitors (FAPs) depletion showed no differences in a) muscle atrophy and b) myofibre cross-sectional area after VML compared to the control (n = 5 mice per condition). c) and d) There were also no differences in hindlimb function with FAP depletion compared to the control groups (n = 5 mice per condition). Data are presented as means. Error bars indicate the SD. Solid line indicates p < 0.05, and dashed lines indicate p < 0.01. TM, Tamoxifen.

Unlike the results in the TT + DN model, FAP depletion had no significant effect on TA muscle weight loss six weeks post-VML compared to controls (-37.62 ± 8.08% vs -30.51 ± 7.20%, p = 0.244; Figure 6a). There was also no significant difference in myofibre size post-VML between the FAP depletion and control groups (Figure 6b). Similarly, hindlimb functional metrics, including mean stride length (2.51 ± 0.89 cm vs 2.73 ± 0.51 cm, p = 0.975; Figure 6c) and mean paw area at peak stance (0.30 ± 0.06 cm^2^ vs 0.34 ± 0.05 cm^2^, p = 0.822; Figure 6d), were unaffected by FAP depletion.

## Discussion

In this study, we have applied an inducible murine FAP depletion model to two clinically relevant injury models of RCT and TA VML. Our results demonstrated that FAP depletion reduces fibrosis and FI after RCT, along with the attenuation of muscle atrophy and improved global shoulder function. Meanwhile, in the TA VML model, FAP depletion decreased muscle fibrosis but did not impact muscle regeneration or functional recovery. These findings highlight distinct roles of FAPs in muscle regeneration, with their contributions varying depending on the clinically specific context and underlying mechanisms.

FAPs have been heavily implicated in muscle degenerative processes. Multiple studies have shown that in chronic injuries, FAPs differentiate into adipocytes and fibroblasts, which directly contribute to post-injury muscle fibrosis and fat accumulation.^3,5,25-27^ Particularly in rotator cuff injuries, the FI from adipogenic FAP differentiation has been linked to poor clinical outcomes and higher re-tear rates.^28-31^ Recent transcriptomic studies have shown that FAPs represent a heterogeneous cell population capable of both regenerative and pathological responses to muscle injury.^15,32^ Malecova et al^33^ found that FAPs contain a distinct, dynamic set of subpopulations that respond differently to acute injury. Garcia et al^8^ have recently demonstrated the presence of at least 11 subpopulations of FAPs in human rotator cuff muscle, which dynamically change following tendon tears. Utilizing an in vivo FAP depletion mouse model, we demonstrated the multipotent role of FAPs in muscle regeneration and degeneration in this study.

More specifically, in both the RCT and TA VML clinical models, we showed that the depletion of FAPs led to a decreased level of muscle fibrosis after injury. Previous studies have suggested that FAPs are the main source of intramuscular fibrosis development across various musculoskeletal injuries.^5,34-37^ While the exact mechanism remains unclear, local signalling post-injury and baseline differences in FAP subpopulations are thought to play a major role in influencing FAPs toward fibroblast differentiation. For example, transforming growth factor-β, secreted from macrophages that are brought to the injury site, has been associated with stimulating FAP differentiation into fibroblasts.^5,35,38^ A subpopulation of FAPs that express the surface marker CD55 has also recently been identified to have upregulation of genes associated with fibrosis and appears to be primed toward eventual fibrotic differentiation.^8^ Thus, our results support the idea that FAPs play a similar and prominent role in post-injury fibrosis across different clinically relevant injury models, and that there may be shared signalling pathways that push FAP fibroblast differentiation. Further, these findings suggest that FAP subpopulations primed for fibrosis may exist similarly in different muscles, such as the supraspinatus and TA.

However, for fatty degeneration, while there was a decrease in the RCT model after FAP depletion, there was no FI seen across any groups for VML. Multiple signalling pathways have been identified that contribute to FAP differentiation in adipocytes which are separate from those that influence FAPs towards fibroblasts.^39-41^ Factors secreted by interleukin-4-polarized macrophages enhance FAP adipocyte differentiation,^40^ while the growth factor, bone morphogenetic protein-7 (BMP-7) also has a similar effect.^42^ Additionally, subpopulations of FAPs marked for eventual adipogenesis, such as with delta-like non-canonical Notch ligand 1, similarly exist and have been suggested to differ between anatomical locations.^8,43^ The differences in FI we observed between the RCT and VML groups could be explained by distinct signalling changes within each clinical context, as the effects of BMP-7 signalling on FAP adipogenic differentiation have been shown in rotator cuff injuries but not yet demonstrated in VML.^42,44^ Unlike what we observed for post-injury fibrosis, differences in the subpopulations of FAPs that are destined for adipogenesis may also be present between the supraspinatus and TA, with FAPs from the latter possibly having less of a role in fatty degeneration after TA VML.

Interestingly, in our massive RCT injury model with both TT + DN, there was functional improvement of the shoulder with FAP depletion. As there was a significant decrease in maladaptive muscle remodelling with FAP depletion, namely decreased fibrosis and FI, this finding could reflect general improvement in shoulder joint mobility and kinematics at six weeks post-TT + DN.^45,46^ Moreover, unlike in humans, surgically transected rotator cuff tendons in mice have been shown to reattach with scar tissue and allow for partial recovery of shoulder function.^47^ Studies in other nerve injury models have demonstrated the ability of injured motor neurones to extend regenerating axons and reoccupy neuromuscular junctions within weeks.^48,49^ Thus, partial reinnervation of rotator cuff muscle with reduced fibrosis and FI with FAP depletion could result in the functional improvements seen. However, the possibility and timeline of reinnervation after TT + DN have not yet been established and warrant further investigation.

This study has several limitations. First, while we analyzed the supraspinatus for our RCT model and the TA for our VML model due to their clinical relevance in these models, we did not assess the differences between disease pathology and muscle-specific properties. Future studies will apply our FAP depletion model to a TT + DN on the TA muscle to determine which factors contribute more heavily to our findings. Additionally, while our FAP depletion model demonstrated significant changes in muscle regeneration outcomes, it did not reduce the entire FAP population. Different tamoxifen induction protocols could increase FAP depletion and further alter post-injury changes.

In summary, we have used an inducible in vivo FAP depletion mouse model to study the roles of FAPs within different clinically relevant contexts. The depletion of FAPs in RCTs attenuates fibrosis, FI, and muscle atrophy with improved global shoulder function. In TA VML, FAP depletion demonstrated decreased fibrosis, but no differences in muscle regeneration or functional outcomes. These findings suggest that FAPs play varying roles in different clinically specific contexts, highlighting the need to study the detailed mechanisms of each injury and muscle type to determine appropriate FAP-targeted treatments.

## Data Availability

The data used and/or analyzed for this study are available from the corresponding author upon reasonable request.
